# Using a Convolutional Neural Network to Predict Remission of Diabetes After Gastric Bypass Surgery: Machine Learning Study From the Scandinavian Obesity Surgery Register

**DOI:** 10.2196/25612

**Published:** 2021-08-19

**Authors:** Yang Cao, Ingmar Näslund, Erik Näslund, Johan Ottosson, Scott Montgomery, Erik Stenberg

**Affiliations:** 1 Clinical Epidemiology and Biostatistics School of Medical Sciences Örebro University Örebro Sweden; 2 Unit of Integrative Epidemiology Institute of Environmental Medicine Karolinska Institutet Stockholm Sweden; 3 Department of Surgery Faculty of Medicine and Health Örebro University Örebro Sweden; 4 Division of Surgery Department of Clinical Sciences Danderyd Hospital, Karolinska Institutet Stockholm Sweden; 5 Clinical Epidemiology Division Department of Medicine Karolinska Institutet Stockholm Sweden; 6 Department of Epidemiology and Public Health University College London London United Kingdom

**Keywords:** forecasting, clinical decision rules, remission induction, type 2 diabetes mellitus, gastric bypass, morbid obesity

## Abstract

**Background:**

Prediction of diabetes remission is an important topic in the evaluation of patients with type 2 diabetes (T2D) before bariatric surgery. Several high-quality predictive indices are available, but artificial intelligence algorithms offer the potential for higher predictive capability.

**Objective:**

This study aimed to construct and validate an artificial intelligence prediction model for diabetes remission after Roux-en-Y gastric bypass surgery.

**Methods:**

Patients who underwent surgery from 2007 to 2017 were included in the study, with collection of individual data from the Scandinavian Obesity Surgery Registry (SOReg), the Swedish National Patients Register, the Swedish Prescribed Drugs Register, and Statistics Sweden. A 7-layer convolution neural network (CNN) model was developed using 80% (6446/8057) of patients randomly selected from SOReg and 20% (1611/8057) of patients for external testing. The predictive capability of the CNN model and currently used scores (DiaRem, Ad-DiaRem, DiaBetter, and individualized metabolic surgery) were compared.

**Results:**

In total, 8057 patients with T2D were included in the study. At 2 years after surgery, 77.09% achieved pharmacological remission (n=6211), while 63.07% (4004/6348) achieved complete remission. The CNN model showed high accuracy for cessation of antidiabetic drugs and complete remission of T2D after gastric bypass surgery. The area under the receiver operating characteristic curve (AUC) for the CNN model for pharmacological remission was 0.85 (95% CI 0.83-0.86) during validation and 0.83 for the final test, which was 9%-12% better than the traditional predictive indices. The AUC for complete remission was 0.83 (95% CI 0.81-0.85) during validation and 0.82 for the final test, which was 9%-11% better than the traditional predictive indices.

**Conclusions:**

The CNN method had better predictive capability compared to traditional indices for diabetes remission. However, further validation is needed in other countries to evaluate its external generalizability.

## Introduction

Bariatric surgery is an efficient and safe treatment for patients with morbid obesity and type 2 diabetes (T2D) [1,2]. In obese patients who also have T2D, more than three-fourths of patients show remission after gastric bypass surgery [3,4]. Although remission rates may differ across different surgical procedures, high remission rates have been reported for Roux-en-Y gastric bypass [1,3]. Despite many patients experiencing remission of diabetes, duration and severity of disease, along with age, have been presented as factors associated with reduced chance of achieving remission [1,5]. Prediction of diabetes remission can be helpful in clinical preoperative consultation and decision-making, and several indices have been constructed for this purpose. Scores like DiaRem [6], Ad-DiaRem [7], DiaBetter [8], and the individualized metabolic surgery (IMS) score [9], as well as the age, body mass index, C-peptide level, and duration of T2D (ABCD) score [10] have been used for predicting diabetes remission after bariatric surgery. Many of the models based on the scores have high predictive capability and may already provide clinical guidance [11]. These tools might be helpful for personalized management of morbidly obese individuals with diabetes when considering bariatric surgery in routine care, ultimately contributing to precision medicine [12]. However, the performance of the scores in various studies is not consistent [7]. Previous prediction models were either limited by small sample sizes or were not validated using external data that were not seen by the models during model construction. Therefore, both the performance and validity of the models or scores need to be further evaluated and improved using a larger bariatric surgery database. In recent years, there have been a number of attempts to use artificial intelligence (AI) algorithms, including support vector machine [13], decision tree [14], random forest [15], and deep learning algorithms, such as artificial neural networks [16,17], to incorporate preoperative predictors in predicting outcomes of bariatric surgery. Compared with the traditional statistical regression models, AI algorithms have shown great promise in the field of bariatric surgery [18,19]. However, to our knowledge, none have thus far reached clinical practice.

The aim of this study was to construct a prediction model for T2D remission using a deep learning AI algorithm (ie, convolutional neural network [CNN)]) and to compare its predictive capability with that of 4 widely used predictive scores.

## Methods

### Study Participants

The study used the data from the Scandinavian Obesity Surgery Register (SOReg), a validated, national quality register covering virtually all bariatric and metabolic surgical procedures in Sweden [20]. By using the unique Swedish personal identification number, we linked SOReg to the Swedish National Patient Register, the Swedish National Death Register, the Swedish Prescribed Drug Register, and Statistics Sweden to obtain information on inpatient and outpatient hospital visits, mortality, dispensed drugs, and individual socioeconomic data. The inclusion criteria for patients registered in the SOReg were included those operated on with a primary Roux-en-Y gastric bypass procedure between 2007 and 2015 and those diagnosed with T2D preoperatively, as defined by the American Diabetes Association (ie, fasting plasma glucose ≥ 126 mg/L [7.0 mmol/L], hemoglobin A1c [HbA_1c_] ≥ 48 mmol/mol [6.5%], or pharmacological treatment for diabetes) [21].

### Outcome and Predictor Variables

The main outcome measure was complete remission of diabetes 2 years after surgery, defined as being without diabetes medication within a time frame of +/- 6 months; that is, 18-30 months postoperatively with normal HbA_1c_ value <42 mmol/mol (6.0%) in accordance with the definitions of the American Diabetes Association [22]. Due to loss of information of HbA_1c_ at follow-up, analyses of a secondary outcome, complete remission, defined as discontinuance of pharmacological treatment from 18-30 months, was performed.

The predictor variables were patients’ demographic and socioeconomic information including age, sex, education level (primary, secondary, higher education <3 years, and high education ≥3 years), and region of residence characteristics (large city, medium city or town, and small town or rural area); preoperative BMI, HbA_1c_, and treatment information including insulin treatment, metformin use, other noninsulin pharmacological treatment, and number of antidiabetic drugs; and preoperative comorbidities including sleep apnea, hypertension, dyslipidemia, depression, and cardiovascular comorbidity.

### Descriptive Analysis

Continuous variables are presented as mean and SD, and ordered and nominal variables are presented as median and interquartile range (IQR) and count and percentage, respectively. For comparison between 2 groups, the *t* test and Mann-Whitney test were used for continuous and ordered variables, respectively, while the Pearson chi-square test was used for categorical variables. A 2-tailed *P* value <.05 was considered to be statistically significant.

### Multiple Imputation for Missing Values

Missing values were assumed missing at random and imputed using a random forest algorithm, which has the desirable properties of being able to handle mixed types of missing data, being adaptive to interactions and nonlinearity, and having the potential to scale to big data settings [23]. To allow for the uncertainty of the imputation, 100 imputed data sets were generated in the current study.

### Data Normalization

Because the range of values of variables varies widely (such as for age and BMI) in some machine learning (ML) algorithms, objective function will not work properly [24]. Therefore, the continuous and ordered variables were normalized to have a mean of 0 and a standardization of 1, and the multicategory nominal variables (education and residence) were converted into several binary variables before they were entered into the ML models [25].

### Predictive Model

In the current study, we used a 7-layer CNN model with two 1D convolution layers (with 100 filters for each), two 1D max pooling layers, one flatten layer, and two dense layers (with 1000 computation units) [26,27]. The rectified linear unit activation function was used for the two 1D convolution layers and the first dense layers, and the sigmoid activation function was used for the last dense layer. The binary cross-entropy loss function and the adaptive moment estimation (Adam) optimizer were used when compiling the model [28].

### Model Training, Validation, and Test

**Figure 1:**
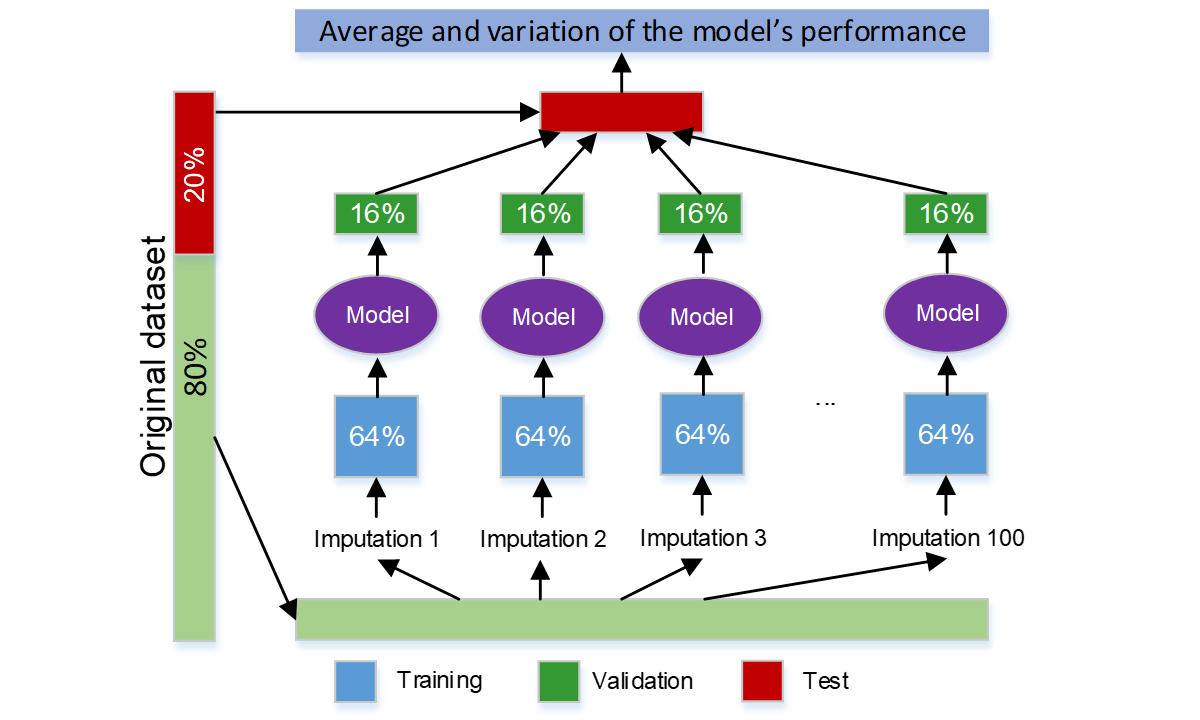
Procedure for training, validation, and testing for the convolutional neural network model.

The whole data set was randomly split into 2 parts: a training data set with 80% (6446/8057) of the patients and a test data set with 20% (1611/8057) of the patients. During the model training stage, the training data set was further divided into 2 data sets: one data set with 64% (5156/8057) of the patients to train the CNN model and another with 16.01% (1290/8057) of the patients to validate the model. Finally, the model was tested using the test data set that was never seen by the CNN model. The CNN model was trained, validated, and tested with the 100 imputations (Figure 1).

### Indices of Predictive Ability

Predictive ability of the CNN model was evaluated using the following indices: area under the receiver operating characteristic (ROC) curve, sensitivity, specificity, and the Youden J [29]. The terminology and derivations of the values have been previously presented in detail [18]. The sensitivity and specificity presented in this study are the values on the ROC curve where the Youden J achieves the maximum value. The acceptable, excellent, and outstanding predictive models were defined as those with an area under the ROC curve (AUC) greater than 0.7, 0.8, and 0.9, respectively [30,31]. The average and the 95% CI of the indices were calculated based on 100 imputations.

### Comparison Between the CNN Model and DiaRem, Ad-DiaRem, DiaBetter, and IMS

We also evaluated the predictive capability of the currently used indices, DiaRem, Ad-DiaRem, DiaBetter, and IMS, and compared them with the CNN model. The DiaRem score is calculated using insulin use, age, HbA_1c_ value, and type of antidiabetic drugs [32]. The Ad-DiaRem score is a modification of the DiaRem score, calculated using insulin use, age, HbA_1c_ value, number of antidiabetic drugs, duration of diabetes, and number of antidiabetic drugs [13]. The DiaBetter is calculated using HbA_1c_, type of antidiabetic drugs, and duration of diabetes [8]. The IMS score is calculated using the number of preoperative diabetes medications, insulin use, duration of diabetes, and HbA_1c_ level [9].

The points on the nonparametric ROC curve of DiaRem, Ad-DiaRem, DiaBetter, and IMS were generated using each value as a classification cutoff point and computing the corresponding sensitivity and one minus specificity. These points were then connected by straight lines, and the AUC was computed using the trapezoidal rule [33].

The same training and testing procedure used for the CNN model was also applied for the 4 scores.

### Software and Hardware

The descriptive analysis and evaluation for DiaRem, Ad-DiaRem, DiaBetter, and IMS were conducted in Stata 16.1 (StataCorp LLC). The CNN model was achieved in Python 3.6 (Python Software Foundation) using the Keras 2.4.0 and Scikit-learn 0.23 packages. All the computation was operated on a computer with 64-bit Windows 7 Enterprise operating system (Service Pack 1, Microsoft Corporation), an Intel Core TM i5-4210U 2.40-GHz CPU, and 16.0 GB installed random access memory.

### Ethics

The study was approved by the regional ethics committee in Stockholm (reference #2013/535-31/5, #2014/1639-32, and #2017/857-32). The study was conducted according to the guidelines of the Transparent Reporting of a Multivariable Prediction Model for Individual Prognosis or Diagnosis (TRIPOD) statement [34].

## Results

### Patient Characteristics

In total, 8112 patients met the inclusion criteria; after exclusion of 55 patients who died within the first 2 years after surgery, 8057 patients remained in the analysis. Information on pharmaceutical usage before and after surgery was available for all patients. A postoperative weight was registered for 7268 patients at 1 year after surgery (90.21%), and 4996 patients at 2 years after surgery (62.01%). A postoperative glycosylated HbA_1c_ test result was available for 6989 patients (86.74%). Baseline characteristics of the included patients are shown in Table 1. Statistically significant differences were found for almost all the predictor variables between the remission patients and nonremission patients, except for depression and education (Table 1), which implies the potential for using the predictor variables to predict outcome. Preoperative HbA_1c_ values were missing for about one-seventh of the patients, indicating the need for imputation since the predictive capability otherwise would be significantly reduced and biased by excluding a considerable proportion of the data with missing values. Patients with a missing HbA_1c_ value were more often males of marginally higher age and longer duration of disease, and small differences were also seen in terms of pharmacological treatment, education, and residence (Supplementary Table S1, Multimedia Appendix 1). After multiple imputation, similar distributions of HbA_1c_ values were seen (Supplementary Figure S1, Multimedia Appendix 1).

**Table 1:** Characteristics of study participants with further stratification on remission of diabetes (N=8057)^a^.

Characteristic			Overall (n=8057)	Nonremission (n=1846)		Remission (n=6211)		*P* value^b^
Age (years), mean (SD)			47.7 (10.1)	51.7 (8.7)		46.6 (10.2)		<.001
**Sex, n (%)**								.001
	Women	4970 (61.68)		1079 (58.45)	3891 (62.65)			
	Men	3087 (38.32)		767 (41.55)	2320 (37.35)			
BMI (kg/m^2^), mean (SD)			42.22 (5.74)	41.16 (5.44)		42.53 (5.80)		<.001
Hemoglobin A_1c_ (mmol/mol) mean, (SD)			59.0 (17.3)	67.4 (17.5)		56.7 (16.5)		<.001
Diabetes duration (years), median (IQR)			2.0 (0.0-6.0)	6.0 (3.0-10.0)		1.0 (0.0-4.0)		<.001
Number of drugs, median (IQR)			1.0 (1.0-2.0)	2.0 (1.0-2.0)		1.0 (0.0-2.0)		<.001
Insulin, n (%)			2313 (28.71)	1184 (64.14)		1129 (18.18)		<.001
Metformin, n (%)			5610 (69.63)	1618 (87.65)		3992 (64.27)		<.001
Other noninsulin treatment, n (%)			1912 (23.73)	745 (40.36)		1167 (18.79)		<.001
Sleep apnea, n (%)			1529 (18.98)	383 (20.75)		1146 (18.45)		.03
Hypertension, n (%)			4546 (56.42)	1287 (69.72)		3259 (52.47)		<.001
Cardiovascular comorbidity, n (%)			917 (11.38)	305 (16.52)		612 (9.85)		<.001
Dyslipidemia, n (%)			2527 (31.36)	864 (46.80)		1663 (26.78)		<.001
Depression, n (%)			1297 (16.10)	311 (16.85)		986 (15.88)		.34
**Education, n (%)**								.40
	Elementary education	1606 (19.93)		392 (21.24)	1214 (19.55)			
	Secondary education	4762 (59.10)		1091 (59.10)	3671 (59.10)			
	Higher education <3 years	838 (10.40)		179 (9.70)	659 (10.61)			
	Higher education >3 years	796 (9.88)		173 (9.35)	623 (10.03)			
**Residence, n (%)**								.001
	Large city	2734 (33.93)		687 (37.22)	2047 (32.96)			
	Medium-sized town	3061 (37.99		671 (36.35)	2390 (38.48)			
	Small town or rural area	2231 (27.69)		487 (26.38)	1744 (28.08)			
DiaRem, median (IQR)			6.0 (3.0-13.0)	16.0 (8.0-18.0)		5.0 (3.0-8.0)		<.001
Ad-DiaRem, median (IQR)			7.0 (5.0-11.0)	12.0 (9.0-15.0)		7.00 (4.0-9.0)		<.001
DiaBetter, median (IQR)			3.0 (1.0-6.0)	7.0 (5.0-8.0)		3.0 (1.0-4.0)		<.001
IMS^c^, median (IQR)			39.8 (16.0-75.2)	87.2 (59.9-107.2)		28.6 (16.0-57.8)		<.001

^a^Including all the baseline variables used in the study.

^b^*P* value comparing remission vs nonremission.

^c^IMS: individualized metabolic surgery.

### Surgical Outcome

The mean BMI loss at 1 year after surgery was 12.2 kg/m^2^ (SD 4.0 kg/m^2^), with an excess BMI loss (100 × [initial BMI – postoperative BMI]/[initial BMI – 25] %) of 74.0% (SD 22.5%), and a total weight loss (100 × weight loss/preoperative weight%) of 28.7% (SD 7.6%). Mean BMI loss at 2 years after surgery was 12.0 kg/m^2^ (SD 4.53 kg/m^2^) with an excess BMI loss of 73.3% (SD 24.4%) and a total weight loss of 28.4% (SD 8.9%).

At 2 years after surgery, 77.09% (6211/8057) of the patients were able to discontinue the pharmacological treatment of T2D, while complete T2D remission was seen in 63.07% (n=4004) of the 6348 patients who had been evaluated for complete remission.

### Predictive Capability of the CNN Model, DiaRem, Ad-DiaRem, DiaBetter, and IMS

**Figure 2:**
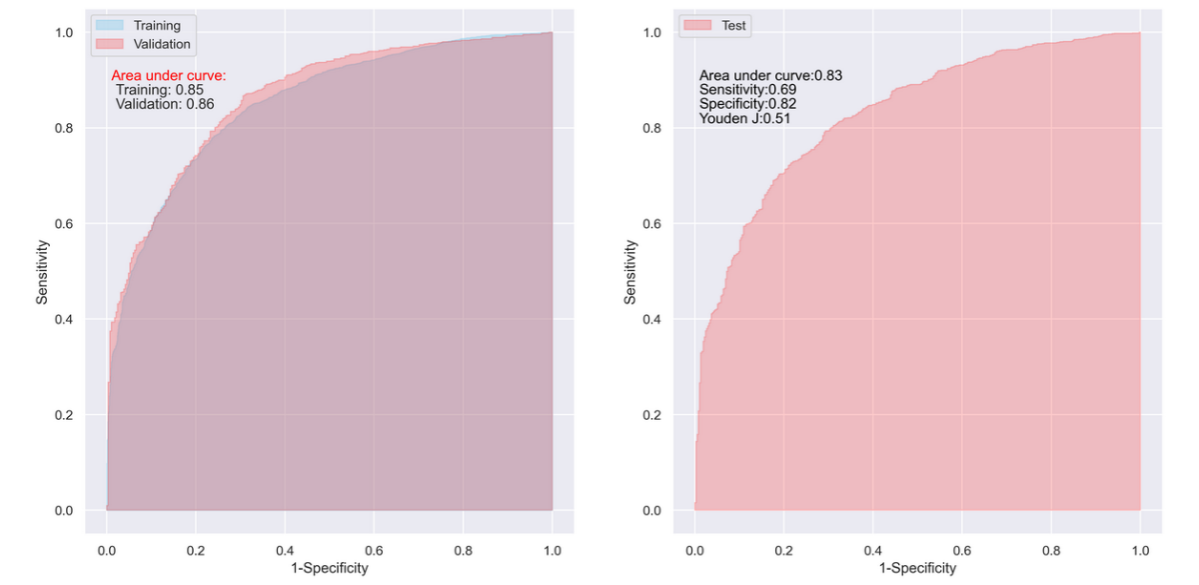
Receiver operating characteristic (ROC) curves of the convolutional neural network model in one of the 100 trainings and validations (left; because the 2 areas under the ROC curves are almost totally overlapping, the blended red and blue colors appear purple), and tests (right).

The predictive capability of the CNN model for the major outcome (remission) is shown in Figure 2 and Table 2. In both the training and validation, the CNN model presented good predictive ability, with an AUC of 0.86 (95% CI 0.85-0.87) and 0.85 (95% CI 0.83-0.86), respectively (Table 2).

**Figure 3:**
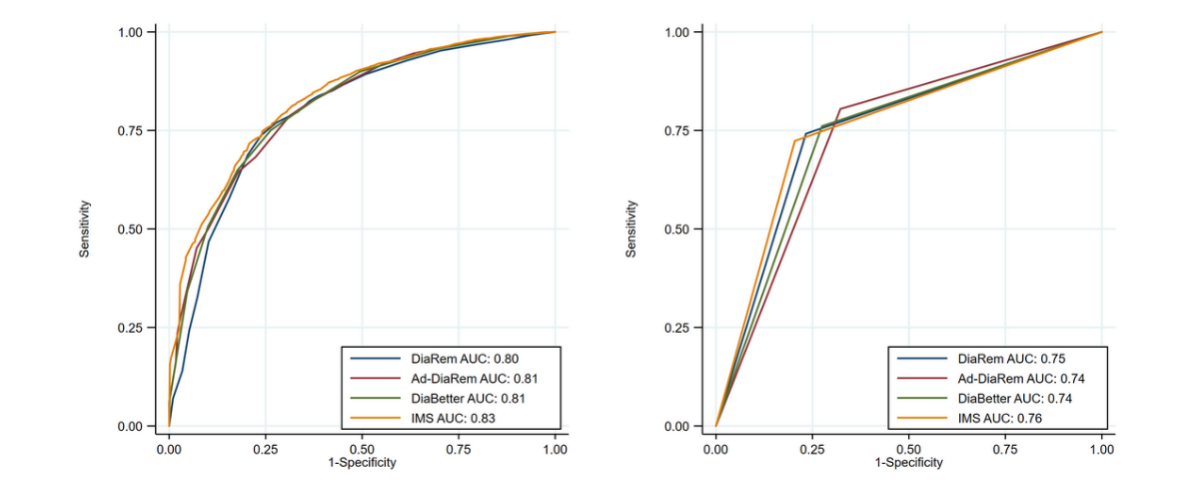
Receiver operating characteristic curves of diabetes indices in one of the 100 trainings (left), and tests (right). AUC: area under the curve.

The DiaRem, Ad-DiaRem, DiaBetter, and IMS also showed good predictive capability in the training with an AUC >0.8 (Figure 3 left and Table 2) but only acceptable predictive ability in the validation (Table 2), with an AUC of 0.73 (95% CI 0.71-0.75), 0.72 (95% CI 0.69-0.74), 0.75 (95% CI 0.72-0.78), and 0.76 (95% CI 0.73-0.79), respectively. In general, the predictive capability of the CNN model was 16.4%, 18.1%, 13.3%, and 11.8% higher than that of DiaRem, Ad-DiaRem, DiaBetter, and IMS, in terms of AUC, respectively. In the tests, the AUC for the predictive ability of the CNN (AUC=0.83; 95% CI 0.82-0.85) model was 10.6%, 12.2%, 12.2%, and 9.2% higher than that of DiaRem (AUC=0.75; 95% CI 0.73-0.76), Ad-DiaRem (AUC=0.74; 95% CI 0.71-0.77), DiaBetter (AUC=0.74; 95% CI 0.72-0.76), and IMS (AUC=0.76; 95% CI 0.73-0.78), respectively (Figure 2 right and Figure 3 right).

**Table 2:** Predictive capability of the CNN model and diabetes indices for the major outcome.

Models by index		Value (95% CI)	
		Training	Validation
**AUC^a^**			
	CNN^b^	0.86 (0.85-0.87)	0.85 (0.83-0.86)
	DiaRem	0.81 (0.79-0.82)	0.73 (0.71-0.75)
	Ad-DiaRem	0.82 (0.81-0.83)	0.72 (0.69-0.74)
	DiaBetter	0.82 (0.81-0.83)	0.75 (0.72-0.78)
	IMS^c^	0.84 (0.83-0.85)	0.76 (0.73-0.79)
**Specificity**			
	CNN	0.78 (0.74-0.83)	0.78 (0.72-0.85)
	DiaRem	0.76 (0.80-0.73)	0.81 (0.78-0.85)
	Ad-DiaRem	0.70 (0.68-0.71)	0.75 (0.70-0.79)
	DiaBetter	0.76 (0.74-0.78)	0.76 (0.71-0.80)
	IMS	0.77 (0.72-0.82)	0.77 (0.72-0.81)
**Sensitivity**			
	CNN	0.77 (0.73-0.82)	0.76 (0.70-0.83)
	DiaRem	0.75 (0.71,0.78)	0.65 (0.62-0.67)
	Ad-DiaRem	0.79 (0.78-0.80)	0.69 (0.67-0.72)
	DiaBetter	0.75 (0.74-0.76)	0.75 (0.72-0.78)
	IMS	0.75 (0.70-0.80)	0.75 (0.73-0.77)
**Youden J**			
	CNN	0.56 (0.54-0.57)	0.54 (0.50-0.59)
	DiaRem	0.51 (0.50-0.52)	0.46 (0.42-0.50)
	Ad-DiaRem	0.48 (0.47-0.49)	0.44 (0.39-0.49)
	DiaBetter	0.51 (.049-0.54)	0.51 (0.45-0.56)
	IMS	0.52 (0.50-0.54)	0.52 (0.47-0.57)

^a^AUC: area under the receiver operating characteristic curve.

^b^CNN: convolutional neural network.

^c^IMS: individualized metabolic surgery.

For the secondary outcome, complete remission, the CNN model also presented a good predictive capability in both the training and validation, with an AUC of 0.84 (95% CI 0.83-0.85) and 0.83 (95% CI 0.81-0.85), respectively (Supplementary Table S4, Multimedia Appendix 1). Although DiaRem, Ad-DiaRem, DiaBetter, and IMS showed good predictive ability in the training with an AUC ≥0.80, they only showed acceptable predictive ability in the validation with an AUC of 0.72 (95% CI 0.69-0.75), 0.72 (95% CI 0.69-0.74), 0.74 (95% CI 0.72-0.77), and 0.74 (95% CI 0.72-0.76), respectively (Supplementary Table S4, Multimedia Appendix 1). In general, the predictive capability of the CNN model was 15.3%, 15.3%, 12.2%, and 12.2% higher than that of DiaRem, Ad-DiaRem, DiaBetter, and IMS, in terms of AUC, respectively.

In the tests, the AUC for the predictive capability of the CNN model (AUC=0.82; 95% CI 0.81-0.83) was 9.3%, 10.8%, 10.8%, and 9.3% higher than that of DiaRem (AUC=0.75; 95% CI 0.73-0.78), Ad-DiaRem (AUC=0.74; 95% CI 0.73-0.75), DiaBetter (AUC=0.74; 95% CI 0.71-0.76), and IMS (AUC=0.75; 95% CI 0.73-0.77), respectively (Supplementary Figure S2 right and S3 right, Multimedia Appendix 1).

## Discussion

### Principal Findings

The CNN model evaluated in this study showed high accuracy for cessation of antidiabetic drugs and complete remission of T2D after gastric bypass surgery, providing 9%-12% better predictive indices compared to available scores.

The currently available and widely accepted predictive indices for diabetes remission, including DiaRem, Ad-DiaRem, DiaBetter, and IMS, were assessed in our study and are all simple and easily available to clinicians for clinical decision support. In addition, one other index, the ABCD score [35], also includes c-peptide. This laboratory measure additionally includes information of endogenous insulin production and could thus potentially further enhance the effectiveness of a prediction model. However, the ABCD score has not been shown to have higher predictive capacity compared to other available models, and it is highly possible that other measures of severity of T2D disease, such as duration of disease, HbA_1c_ value, and type and number of drugs, may provide the same or even better measures for a prediction model [11].

The main benefits of the CNN method, in comparison to the scores based on traditional statistical methods, lie in its ability to include a high number of variables and to learn over time. In contrast to available models designed to offer simple entry and calculations of the most important variables, it offers the ability to handle variables in a more complex way, also including variables of smaller impact. Furthermore, the model construction is not limited by the statistical assumptions and distribution of the data, which usually need to be fulfilled in the traditional regression methods. Exposing the AI to a higher quantity of real-world data also has the potential to further improve it with cumulative learning.

### Implications

The use of AI or machine learning techniques in medical research and practice is currently an evolving field with great potential. Although the exact role of AI in this setting remains to be established, one potential area where the AI seems to outperform traditional techniques is indeed in the construction of prediction models for outcomes from surgical procedures [36]. Previous studies on the construction of prediction models for perioperative complications have reported discouraging results, mainly as a direct consequence of the complexity and diversity of causes for perioperative complications [18,27,37]. In contrast with safety outcomes, efficacy outcomes (in particular those of highly standardized surgical methods such as gastric bypass) may be more suited for adequate prediction models since the factors influencing long-term effects are less diverse. Remission of diabetes is one such outcome that is largely influenced by a few specific factors, making prediction models more easily available. The results of our study support the promising results from previous studies with smaller sample sizes using sparse support vector machine, decision tree, and artificial neural networks to predict diabetes remission after bariatric surgery [13,14,17].

Although our CNN model did not include postoperative weight loss, a factor known to be associated with higher remission and reduced relapse of diabetes [8], the model included measures of patient-specific characteristics, information on duration and severity of disease, and a few socioeconomic factors that all should be easily available at the time of consultation before surgery. Although it is likely that the model could have reached a higher precision if postoperative results (such as early weight loss or improvement in glucose homeostasis) were included, these measures are not available in the preoperative setting and their inclusion would therefore reduce the clinical usefulness of the model [1,5,38]. Age, duration of diabetes, preoperative HbA_1c_, and diabetes medications are all known predictive factors [1,5]. In addition, the model identified sex, BMI, metabolic and cardiovascular comorbidities, and place of residence as factors influencing the chance of diabetes remission.

Although the disposition of adiposity and insulin resistance appears to affect men and women differently [39], differences between sexes may be highly influenced by other covarying factors, such as obesity-related comorbidities, BMI, and age [1]. Indeed, when adjustment is made for other factors, the influence of sex on outcome tends to shift [1]. The influence of BMI on remission rates is also controversial [40]. Patients with higher BMI may have a greater degree of insulin resistance and a higher expected total weight loss [41,42], and may thus benefit more from the favorable metabolic effect of bariatric surgery. However, the influence of BMI on remission can be related to several other factors of relevance for both diabetes remission and postoperative weight loss. Whether or not the influence of BMI is strictly weight dependent or not remains to be answered. Although no difference in remission dependent on educational level was seen, place of residence was associated with the chance of achieving diabetes remission. Residents of larger cities may experience higher life stress and represent a more diverse socioeconomic population [43]. Many socioeconomic factors (such as education, income, profession, and ethnicity) have been reported to influence other efficacy outcomes, such as weight loss, which in turn may contribute to these differences [42].

### Challenges and Limitations

In contrast to traditional regression models, we observed significant improvement with the continuous training process. When increasing amounts of data in the test data set were seen by the CNN model (or more data in the test data set leaked into the training data set), AUC, specificity, and sensitivity increased gradually and eventually approximated 1 (Supplementary Figure S5, Multimedia Appendix 1). From training with more available data and decorrelating data with methods such as principal component analysis, the predictive capability of the CNN model could be improved even further, at least in the Swedish context. To generalize the application of the CNN model, a multinational registration consortium of gastric bypass surgery patients would be needed for improved model training and validation. However, the capacity of memory is also a limitation of the CNN because it reduces the model’s flexibility to incorporate the information from external unseen data, which results in overfitting to specific past data or underfitting to the new data and impedes generalization of the model [44]. Teaching neural networks to strategically forget is an important task in ML. This highlights one of the major challenge of ML techniques [45]. To fulfill this task, incorporating long short-term memory units into CNN networks has been attempted to process temporal sequences and reduce model parameters in human face and activity recognition, which has shown consistent superior performance and good generalization [46,47]. Furthermore, the methods of ML are less transparent and more complex than those of traditional regression models, making their exact nature more difficult to scrutinize [44]. In the absence of clear guidelines, we have—to the best of our ability—conducted and reported the study to match the requirements of the TRIPOD statement and suggested modifications [34]. The programming code of the study is available at the repository figshare website [48]. Furthermore, the study was only based on data from a single country. For full use of the model, external validation would also be needed in other parts of the world.

Finally, only Roux-en-Y gastric bypass procedures were included in the model. The effects of sleeve gastrectomy on diabetes remission may be expected to differ [40], and thus the model is presently only suited for gastric bypass surgery. Including other surgical methods in future development of the model would further improve generalizability.

Despite these limitations, the CNN model outperformed the currently available high-quality prediction models. It also demonstrated better predictive ability than that mentioned in a previous report on AI for diabetes remission [49]. The CNN model may therefore find a place in the preoperative setting for surgeons, bariatricians, or endocrinologists looking to quantify the probability of diabetes remission in their decision-making for bariatric surgery in a given patient. After further validation, the AI model could be made available on a webpage or as a mobile app to allow user-friendly and fully available use in the clinical context.

### Conclusions

Our CNN-based ML model performed well in identifying morbidly obese patients with T2D who might benefit from Roux-en-Y gastric bypass surgery. We also demonstrated the model had better predictive capability compared with the current widely used 4 comprehensive indices for diabetes remission after gastric bypass surgery. Prospectively identifying this subset of patients using data available at the time of preoperative evaluation provides an opportune time window to intervene and prevent or reduce the risk of morbidity and mortality, and may potentially reduce the total cost of care. However, this model should be further validated in future research using external data in other countries before it is incorporated into clinical practice.

## Appendix

Multimedia Appendix 1 Supplementary materials.

## Abbreviations

ABCD: age, body mass index, C-peptide level, and duration of type 2 diabetes
AI: artificial intelligence
AUC: area under the receiver operating characteristic curve
CNN: convolutional neural network
HbA_1c_: hemoglobin A_1c_
IMS: individualized metabolic surgery
ML: machine learning
SOReg: Scandinavian Obesity Surgery Registry
T2D: type 2 diabetes
TRIPOD: Transparent Reporting of a Multivariable Prediction Model for Individual Prognosis or Diagnosis

## References

[1] Jans A, Näslund I, Ottosson J, Szabo E, Näslund E, Stenberg E (2019). Duration of type 2 diabetes and remission rates after bariatric surgery in Sweden 2007-2015: A registry-based cohort study. PLoS Med.

[2] Sjöström L, Peltonen M, Jacobson P, Ahlin S, Andersson-Assarsson J, Bouchard C, Carlsson B, Karason K, Lönroth H, Näslund I, Sjöström E, Taube M, Wedel H, Svensson P, Sjöholm K, Carlsson LMS, Anveden (2014). Association of bariatric surgery with long-term remission of type 2 diabetes and with microvascular and macrovascular complications. JAMA.

[3] Buchwald H, Estok R, Fahrbach K, Banel D, Jensen MD, Pories WJ, Bantle JP, Sledge I (2009). Weight and type 2 diabetes after bariatric surgery: systematic review and meta-analysis. Am J Med.

[4] Hofsø D, Fatima F, Borgeraas H, Birkeland KI, Gulseth HL, Hertel JK, Johnson LK, Lindberg M, Nordstrand N, Cvancarova Småstuen M, Stefanovski D, Svanevik M, Gretland Valderhaug T, Sandbu R, Hjelmesæth J (2019). Gastric bypass versus sleeve gastrectomy in patients with type 2 diabetes (Oseberg): a single-centre, triple-blind, randomised controlled trial. Lancet Diabetes Endocrinol.

[5] Arterburn DE, Bogart A, Sherwood NE, Sidney S, Coleman KJ, Haneuse S, O'Connor PJ, Theis MK, Campos GM, McCulloch D, Selby J (2013). A multisite study of long-term remission and relapse of type 2 diabetes mellitus following gastric bypass. Obes Surg.

[6] Craig Wood G, Horwitz D, Still CD, Mirshahi T, Benotti P, Parikh M, Hirsch AG (2018). Performance of the DiaRem score for predicting diabetes remission in two health systems following bariatric surgery procedures in Hispanic and non-Hispanic White patients. Obes Surg.

[7] Dicker D, Golan R, Aron-Wisnewsky J, Zucker J, Sokolowska N, Comaneshter DS, Yahalom R, Vinker S, Clément K, Rudich A (2019). Prediction of long-term diabetes remission after RYGB, sleeve gastrectomy, and adjustable gastric banding using DiaRem and Advanced-DiaRem scores. Obes Surg.

[8] Pucci A, Tymoszuk U, Cheung WH, Makaronidis JM, Scholes S, Tharakan G, Elkalaawy M, Guimaraes M, Nora M, Hashemi M, Jenkinson A, Adamo M, Monteiro MP, Finer N, Batterham RL (2018). Type 2 diabetes remission 2 years post Roux-en-Y gastric bypass and sleeve gastrectomy: the role of the weight loss and comparison of DiaRem and DiaBetter scores. Diabet Med.

[9] Aminian A, Brethauer SA, Andalib A, Nowacki AS, Jimenez A, Corcelles R, Hanipah ZN, Punchai S, Bhatt DL, Kashyap SR, Burguera B, Lacy AM, Vidal J, Schauer PR (2017). Individualized metabolic surgery score: procedure selection based on diabetes severity. Ann Surg.

[10] Chen J, Hsu N, Lee W, Chen S, Ser K, Lee Y (2018). Prediction of type 2 diabetes remission after metabolic surgery: a comparison of the individualized metabolic surgery score and the ABCD score. Surg Obes Relat Dis.

[11] Sjöholm K, Carlsson LMS, Taube M, le Roux CW, Svensson P, Peltonen M (2020). Comparison of preoperative remission scores and diabetes duration alone as predictors of durable type 2 diabetes remission and risk of diabetes complications after bariatric surgery: a post hoc analysis of participants from the Swedish obese subjects study. Diabetes Care.

[12] Koliaki C, Tzeravini E, Papachristoforou E, Severi I, El Deik E, Karaolia M, Noutsou M, Thanopoulou A, Kountouri A, Balampanis K, Lambadiari V, Tentolouris N, Kokkinos A (2020). Eligibility and awareness regarding metabolic surgery in patients with type 2 diabetes mellitus in the real-world clinical setting; estimate of possible diabetes remission. Front Endocrinol (Lausanne).

[13] Aron-Wisnewsky J, Sokolovska N, Liu Y, Comaneshter DS, Vinker S, Pecht T, Poitou C, Oppert J, Bouillot J, Genser L, Dicker D, Zucker J, Rudich A, Clément K (2017). The advanced-DiaRem score improves prediction of diabetes remission 1 year post-Roux-en-Y gastric bypass. Diabetologia.

[14] Hayes MT, Hunt LA, Foo J, Tychinskaya Y, Stubbs RS (2011). A model for predicting the resolution of type 2 diabetes in severely obese subjects following Roux-en Y gastric bypass surgery. Obes Surg.

[15] Razzaghi T, Safro I, Ewing J, Sadrfaridpour E, Scott JD (2019). Predictive models for bariatric surgery risks with imbalanced medical datasets. Ann Oper Res.

[16] Thomas DM, Kuiper P, Zaveri H, Surve A, Cottam D (2017). Neural networks to predict long-term bariatric surgery outcomes. Bariatric Times.

[17] Pedersen HK, Gudmundsdottir V, Pedersen MK, Brorsson C, Brunak S, Gupta R (2016). Ranking factors involved in diabetes remission after bariatric surgery using machine-learning integrating clinical and genomic biomarkers. NPJ Genom Med.

[18] Cao Y, Fang X, Ottosson J, Näslund E, Stenberg E (2019). A comparative study of machine learning algorithms in predicting severe complications after bariatric surgery. J Clin Med.

[19] Johnston SS, Morton JM, Kalsekar I, Ammann EM, Hsiao C, Reps J (2019). Using machine learning applied to real-world healthcare data for predictive analytics: an applied example in bariatric surgery. Value Health.

[20] Hedenbro JL, Näslund E, Boman L, Lundegårdh G, Bylund A, Ekelund M, Laurenius A, Möller P, Olbers T, Sundbom M, Ottosson J, Näslund I (2015). Formation of the Scandinavian Obesity Surgery Registry, SOReg. Obes Surg.

[21] American Diabetes Association (2014). Diagnosis and classification of diabetes mellitus. Diabetes Care.

[22] Buse JB, Caprio S, Cefalu WT, Ceriello A, Del Prato S, Inzucchi SE, McLaughlin S, Phillips GL, Robertson RP, Rubino F, Kahn R, Kirkman MS (2009). How do we define cure of diabetes?. Diabetes Care.

[23] Tang F, Ishwaran H (2017). Random forest missing data algorithms. Stat Anal Data Min.

[24] Zheng A, Casari A (2018). Feature Engineering for Machine Learning: Principles and Techniques for Data Scientists.

[25] Lantz B (2019). Machine Learning with R: Expert Techniques for Predictive Modeling.

[26] Ketkar N (2017). Convolutional Neural Networks. Deep Learning with Python.

[27] Cao Y, Montgomery S, Ottosson J, Näslund E, Stenberg E (2020). Deep learning neural networks to predict serious complications after bariatric surgery: analysis of Scandinavian Obesity Surgery Registry data. JMIR Med Inform.

[28] Kingma D, Ba J (2014). Adam: A method for stochastic optimization. arXiv preprint arXiv.

[29] Yin J, Tian L (2014). Joint confidence region estimation for area under ROC curve and Youden index. Stat Med.

[30] Marzban C (2004). The ROC curve and the area under it as performance measures. Weather Forecast.

[31] Mandrekar JN (2010). Receiver operating characteristic curve in diagnostic test assessment. J Thorac Oncol.

[32] Still CD, Wood GC, Benotti P, Petrick AT, Gabrielsen J, Strodel WE, Ibele A, Seiler J, Irving BA, Celaya MP, Blackstone R, Gerhard GS, Argyropoulos G (2014). Preoperative prediction of type 2 diabetes remission after Roux-en-Y gastric bypass surgery: a retrospective cohort study. Lancet Diabetes Endocrinol.

[33] StataCorp LLC (2007). Stata Base Reference Manual Release.

[34] Collins GS, Reitsma JB, Altman DG, Moons KGM, TRIPOD Group (2015). Transparent reporting of a multivariable prediction model for individual prognosis or diagnosis (TRIPOD): the TRIPOD statement. The TRIPOD Group. Circulation.

[35] Lee W, Hur KY, Lakadawala M, Kasama K, Wong SKH, Chen S, Lee Y, Ser K (2013). Predicting success of metabolic surgery: age, body mass index, C-peptide, and duration score. Surg Obes Relat Dis.

[36] Hashimoto DA, Rosman G, Rus D, Meireles OR (2018). Artificial intelligence in surgery: promises and perils. Ann Surg.

[37] Geubbels N, de Brauw LM, Acherman YIZ, van de Laar AWJM, Bruin SC (2015). Risk stratification models: how well do they predict adverse outcomes in a large Dutch bariatric cohort?. Obes Surg.

[38] Yan Y, Wang G, Xu N, Wang F (2014). Correlation between postoperative weight loss and diabetes mellitus remission: a meta-analysis. Obes Surg.

[39] Geer EB, Shen W (2009). Gender differences in insulin resistance, body composition, and energy balance. Gend Med.

[40] Rubino F, Nathan DM, Eckel RH, Schauer PR, Alberti KGMM, Zimmet PZ, Del Prato S, Ji L, Sadikot SM, Herman WH, Amiel SA, Kaplan LM, Taroncher-Oldenburg G, Cummings DE, Delegates of the 2nd Diabetes Surgery Summit (2016). Metabolic surgery in the treatment algorithm for type 2 diabetes: a joint statement by international diabetes organizations. Diabetes Care.

[41] Martinez KE, Tucker LA, Bailey BW, LeCheminant JD (2017). Expanded normal weight obesity and insulin resistance in US adults of the National Health and Nutrition Examination Survey. J Diabetes Res.

[42] Stenberg E, Näslund I, Persson C, Szabo E, Sundbom M, Ottosson J, Näslund E (2020). The association between socioeconomic factors and weight loss 5 years after gastric bypass surgery. Int J Obes (Lond).

[43] Zarzycka D, Ślusarska B, Marcinowicz L, Wrońska I, Kózka M (2014). Assessment of differences in psychosocial resources and state of health of rural and urban residents--based on studies carried out on students during examination stress. Ann Agric Environ Med.

[44] Collins GS, Moons KGM (2019). Reporting of artificial intelligence prediction models. Lancet.

[45] Bourtoule L, Chandrasekaran V, Choquette-Choo C, Jia H, Travers A, Zhang B (2019). Machine unlearning. arXiv preprint arXiv.

[46] Xu Z, Li S, Deng W (2015). Learning temporal features using LSTM-CNN architecture for face anti-spoofing.

[47] Xia K, Huang J, Wang H (2020). LSTM-CNN architecture for human activity recognition. IEEE Access.

[48] Cao Y, Näslund I, Näslund E, Ottosson J, Montgomery S, Stenberg E Python code for: Using convolutional neural network to predict remission of diabetes after gastric bypass surgery – a machine learning study from the Scandinavian Obesity Surgery Register 2020. figshare.

[49] Johnston SS, Morton JM, Kalsekar I, Ammann EM, Hsiao C, Reps J (2019). Using machine learning applied to real-world healthcare data for predictive analytics: an applied example in bariatric surgery. Value Health.

