# Supplementary material for: Using a Convolutional Neural Network to Predict Remission of Diabetes After Gastric Bypass Surgery: Machine Learning Study From the Scandinavian Obesity Surgery Register
Source: JMIR Med Inform. 2021 Aug 19;9(8):e25612. doi: 10.2196/25612 (PMC8414302; doi:10.2196/25612)
Supplement: Multimedia Appendix 1 [file medinform_v9i8e25612_app1.docx]

## Supplemental materials:

| **Supplementary Table S1. Characteristics of study participants stratification on availability of preoperative HbA1c analysis** | | | |
| --- | --- | --- | --- |
|  | Available HbA1c | Non-available HbA1c | p |
| Number of indviduals, n | 6989 | 1068 |  |
| Age, mean ± SD, yrs | 47.7 ± 10.1 | 48.0 ± 10.0 | 0.428 |
| Sex, n (%) |  |  |  |
| Women | 4343 (62.1) | 627 (58.7) | 0.034 |
| Men | 2646 (37.9) | 441 (41.3) |  |
| Body Mass Index, mean ± SD, kg/m^2^ | 42.20 ± 5.74 | 42.31 ± 5.75 | 0.576 |
| Diabetesduration, median [IQR], yrs | 2.0 [0.0, 6.0] | 3.0 [1.0, 6.0] | <0.001 |
| Number of drugs, median [IQR] | 1.0 [1.0, 2.0] | 1.0 [1.0, 2.0] | <0.001 |
| Insulin, n (%) | 1915 (27.4) | 398 (37.3) | <0.001 |
| Metformin, n (%) | 4740 (67.8) | 870 (81.5) | <0.001 |
| Other noninsulin treatment, n (%) | 1597 (22.9) | 315 (29.5) | <0.001 |
| Sleepapnoea, n (%) | 1323 (18.9) | 206 (19.3) | 0.813 |
| Hypertension, n (%) | 3963 (56.7) | 583 (54.6) | 0.206 |
| Cardiovascular comorbidity, n (%) | 779 (11.1) | 138 (12.9) | 0.099 |
| Dyslipidaemia, n (%) | 2196 (31.4) | 331 (31.0) | 0.806 |
| Depression, n (%) | 1132 (16.2) | 165 (15.4) | 0.566 |
| Education, n (%) |  |  | 0.034 |
| Elementary education | 4090 (58.5) | 672 (62.9) |  |
| Secondary education | 1398 (20.0) | 208 (19.5) |  |
| Higher education <3yrs | 744 (10.6) | 94 (8.8) |  |
| Higher education >3 yrs | 710 (10.2) | 86 (8.1) |  |
| Residence, n (%) |  |  | <0.001 |
| Large city | 2481 (35.5) | 253 (23.7) |  |
| Medium-sized town | 2718 (38.9) | 343 (32.1) |  |
| Small town or rural area | 1762 (25.2) | 469 (43.9) |  |

| **Supplementary Table S2. Characteristics of the patients having complete remission information vs. without complete remission information** | | | |
| --- | --- | --- | --- |
| Variable | Information available | Information missing | p |
| Number of indviduals, n | 6438 | 1674 |  |
| Age, mean ± SD, yrs | 48.3 ± 10.0 | 46.0 ± 10.4 | <0.001 |
| **Sex, n (%)** |  |  | 0.002 |
| Women | 4022 (62.5) | 976 (58.3) |  |
| Men | 2416 (37.5) | 698 (41.7) |  |
| Body Mass Index, mean ± SD, kg/m^2^ | 42.2 ± 5.7 | 42.3 ± 5.8 | 0.622 |
| B-HbA1c mean ± SD, mmol/mol | 59.00 ± 17.3 | 59.2 ± 17.6 | 0.752 |
| Diabetesduration, median [IQR], yrs | 2.0 [0.0, 6.0] | 2.0 [0.0, 5.0] | <0.001 |
| Number of drugs, median [IQR] | 1.0 [1.0, 2.0] | 1.00 [1.0, 2.0] | 0.048 |
| Insulin, n (%) | 1861 (28.9) | 469 (28.0) | 0.492 |
| Metformin, n (%) | 4501 (69.9) | 1147 (68.5) | 0.472 |
| Other noninsulin treatment, n (%) | 1559 (24.2) | 363 (21.7) | 0.083 |
| Sleepapnoea, n (%) | 1240 (19.3) | 307 (18.3) | 0.412 |
| Hypertension, n (%) | 3742 (58.1) | 841 (50.2) | <0.001 |
| Cardiovascular comorbidity, n (%) | 746 (11.6) | 190 (11.4) | 0.820 |
| Dyslipidaemia, n (%) | 2090 (32.5) | 461 (27.5) | <0.001 |
| Depression, n (%) | 982 (15.3) | 330 (19.7) | <0.001 |
| **Education, n (%)** |  |  | 0.002 |
| Elementary education | 3816 (59.3) | 969 (57.9) |  |
| Secondary education | 1244 (19.3) | 381 (22.8) |  |
| Higher education <3yrs | 692 (10.7) | 152 ( 9.1) |  |
| Higher education >3 yrs | 647 (10.0) | 154 ( 9.2) |  |
| **Residence, n (%)** |  |  | <0.001 |
| Large city | 2242 (34.8) | 504 (30.1) |  |
| Medium-sized town | 2411 (37.4) | 666 (39.8) |  |
| Small town or rural area | 1764 (27.4) | 480 (28.7) |  |
| DiaRem, median [IQR] | 6.0 [3.0, 14.0] | 5.0 [3.0, 10.0] | <0.001 |
| Ad-DiaRem, median [IQR] | 7.0 [5.0, 11.0] | 7.0 [4.0, 10.0] | <0.001 |
| DiaBetter, median [IQR] | 3.0 [1.0, 6.0] | 3.0 [1.0, 5.0] | <0.001 |
| IMS, median [IQR] | 39.8 [16.0, 76.0] | 34.2 [16.0, 64.8] | <0.001 |

| **Supplementary Table S3. Characteristics of the patients complete remission vs. non complete remission** | | | |
| --- | --- | --- | --- |
| Variable | No | Yes | p |
| Number of indviduals, n | 2434 | 4004 |  |
| Age, mean ± SD, yrs | 51.42 (8.59) | 46.32 (10.22) | <0.001 |
| **Sex, n (%)** |  |  | 0.136 |
| Women | 1492 (61.3) | 2530 (63.2) |  |
| Men | 942 (38.7) | 1474 (36.8) |  |
| Body Mass Index, mean ± SD, kg/m^2^ | 41.16 (5.32) | 42.85 (5.88) | <0.001 |
| B-HbA1c mean ± SD, mmol/mol | 66.07 (17.37) | 54.85 (15.83) | <0.001 |
| Diabetesduration, median [IQR], yrs | 5.0 [2.0, 9.0] | 1.0 [0.0, 4.0] | <0.001 |
| Number of drugs, median [IQR] | 2.0 [1.0, 2.0] | 1.0 [0.0, 1.0] | <0.001 |
| Insulin, n (%) | 1306 (53.7) | 555 (13.9) | <0.001 |
| Metformin, n (%) | 1991 (81.8) | 2510 (62.7) | <0.001 |
| Other noninsulin treatment, n (%) | 936 (38.5) | 623 (15.6) | <0.001 |
| Sleepapnoea, n (%) | 494 (20.3) | 746 (18.6) | 0.107 |
| Hypertension, n (%) | 1657 (68.1) | 2085 (52.1) | <0.001 |
| Cardiovascular comorbidity, n (%) | 369 (15.2) | 377 ( 9.4) | <0.001 |
| Dyslipidaemia, n (%) | 1090 (44.8) | 1000 (25.0) | <0.001 |
| Depression, n (%) | 397 (16.3) | 585 (14.6) | 0.071 |
| **Education, n (%)** |  |  | 0.037 |
| Elementary education | 1453 (59.7) | 2363 (59.0) |  |
| Secondary education | 503 (20.7) | 741 (18.5) |  |
| Higher education <3yrs | 233 (9.6) | 459 (11.5) |  |
| Higher education >3 yrs | 230 (9.4) | 417 (10.4) |  |
| **Residence, n (%)** |  |  | 0.268 |
| Large city | 883 (36.3) | 1359 (33.9) |  |
| Medium-sized town | 885 (36.4) | 1526 (38.1) |  |
| Small town or rural area | 659 (27.1) | 1105 (27.6) |  |
| DiaRem, median [IQR] | 14.0 [6.0, 17.0] | 4.0 [2.0, 7.0] | <0.001 |
| Ad-DiaRem, median [IQR] | 11.0 [8.0, 14.0] | 6.0 [3.0, 9.0] | <0.001 |
| DiaBetter, median [IQR] | 6.0 [4.0, 8.0] | 2.0 [1.0, 4.0] | <0.001 |
| IMS, median [IQR] | 77.4 [45.4, 102.6] | 27.2 [12.6, 48.6] | <0.001 |

| **Supplementary Table S4. Predictive capability of the CNN model and diabetes indices for complete remission** | | | |
| --- | --- | --- | --- |
| Index | Model | Value (95% CI) | |
|  |  | Training | Validation |
| AUC | CNN | 0.84 (0.83, 0.85) | 0.83 (0.81, 0.85) |
|  | DiaRem | 0.80 (0.79, 0.81) | 0.72 (0.69, 0.75) |
|  | Ad-DiaRem | 0.81 (0.80, 0.82) | 0.72 (0.69, 0.74) |
|  | DiaBetter | 0.80 (0.79, 0.81) | 0.74 (0.72, 0.77) |
|  | IMS | 0.81 (0.79, 0.82) | 0.74 (0.72, 0.76) |
| Specificity | CNN | 0.76 (0.71, 0.80) | 0.76 (0.68, 0.83) |
|  | DiaRem | 0.70 (0.65, 0.75) | 0.72 (0.68, 0.76) |
|  | Ad-DiaRem | 0.71 (0.59, 0.84) | 0.75 (0.70, 0.79) |
|  | DiaBetter | 0.67 (0.66, 0.69) | 0.68 (0.64, 0.71) |
|  | IMS | 0.72 (0.68, 0.76) | 0.72 (0.68, 0.76) |
| Sensitivity | CNN | 0.78 (0.74, 0.83) | 0.77 (0.70, 0.84) |
|  | DiaRem | 0.77 (0.73, 0.80) | 0.73 (0.69, 0.76) |
|  | Ad-DiaRem | 0.75 (0.63, 0.87) | 0.69 (0.67, 0.72) |
|  | DiaBetter | 0.81 (0.80, 0.82) | 0.81 (0.78, 0.84) |
|  | IMS | 0.77 (0.73, 0.81) | 0.77 (0.74, 0.80) |
| Youden J | CNN | 0.54 (0.53, 0.55) | 0.53 (0.49, 0.57) |
|  | DiaRem | 0.47 (0.45, 0.49) | 0.44 (0.39, 0.50) |
|  | Ad-DiaRem | 0.46 (0.45, 0.48) | 0.44 (0.39, 0.49) |
|  | DiaBetter | 0.48 (0.46, 0.50) | 0.48 (0.43, 0.53) |
|  | IMS | 0.49 (0.47, 0.50) | 0.48 (0.43, 0.53) |


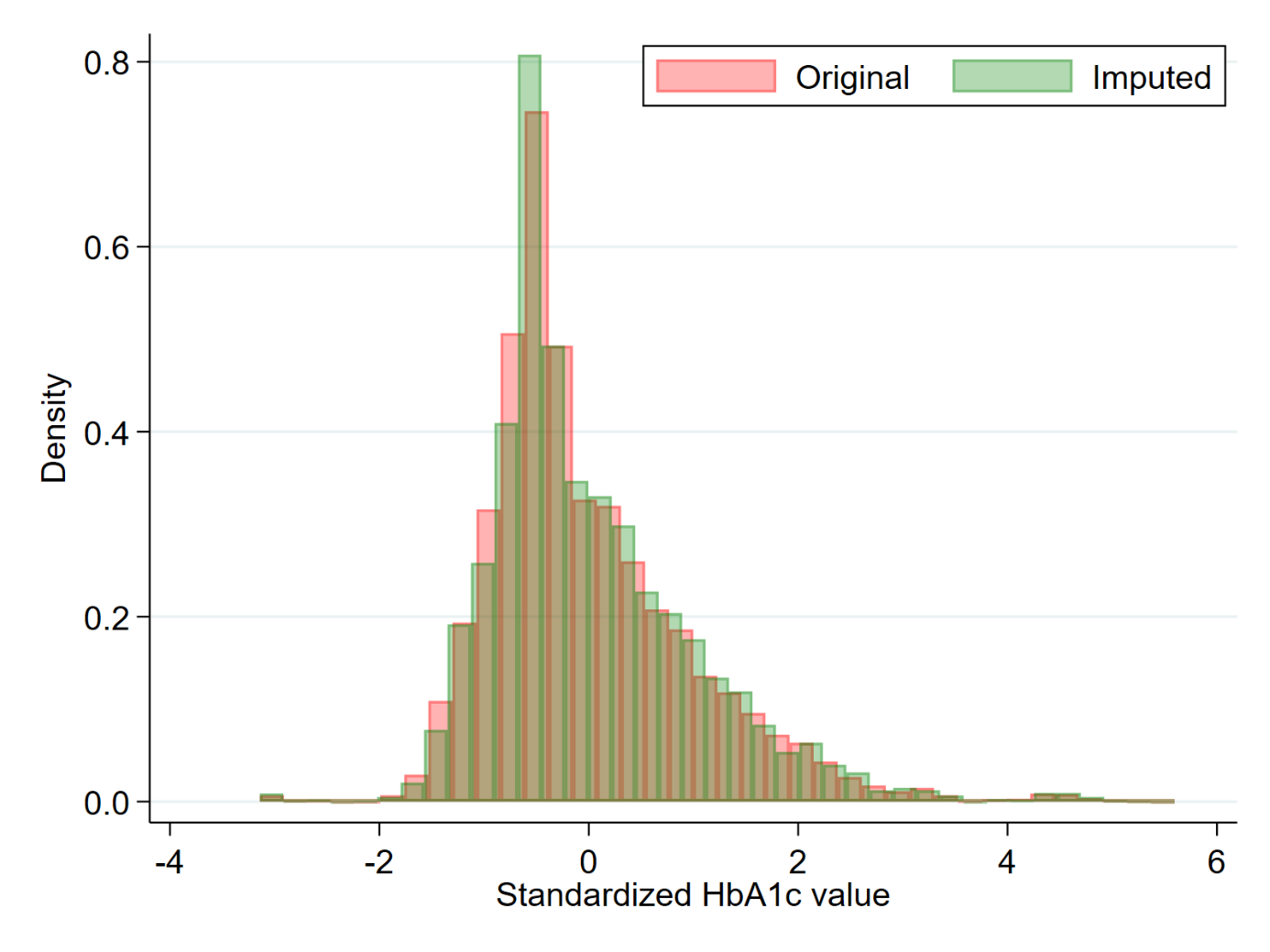


Supplementary Figure S1. Distributions of the original HbA1c values and the HbA1c values after imputation


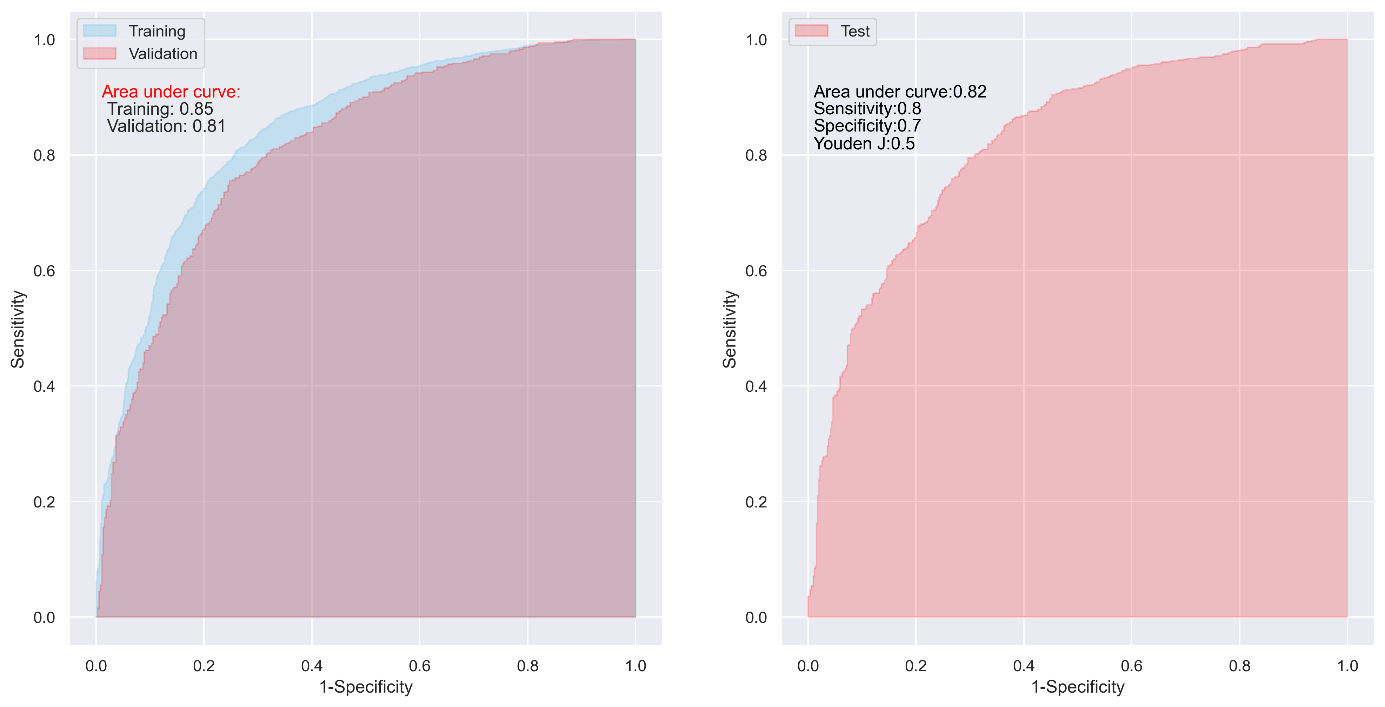


Supplementary Figure S2. ROC curves of the CNN model in one of 100 trainings and validations (left), and tests (right) for complete remission


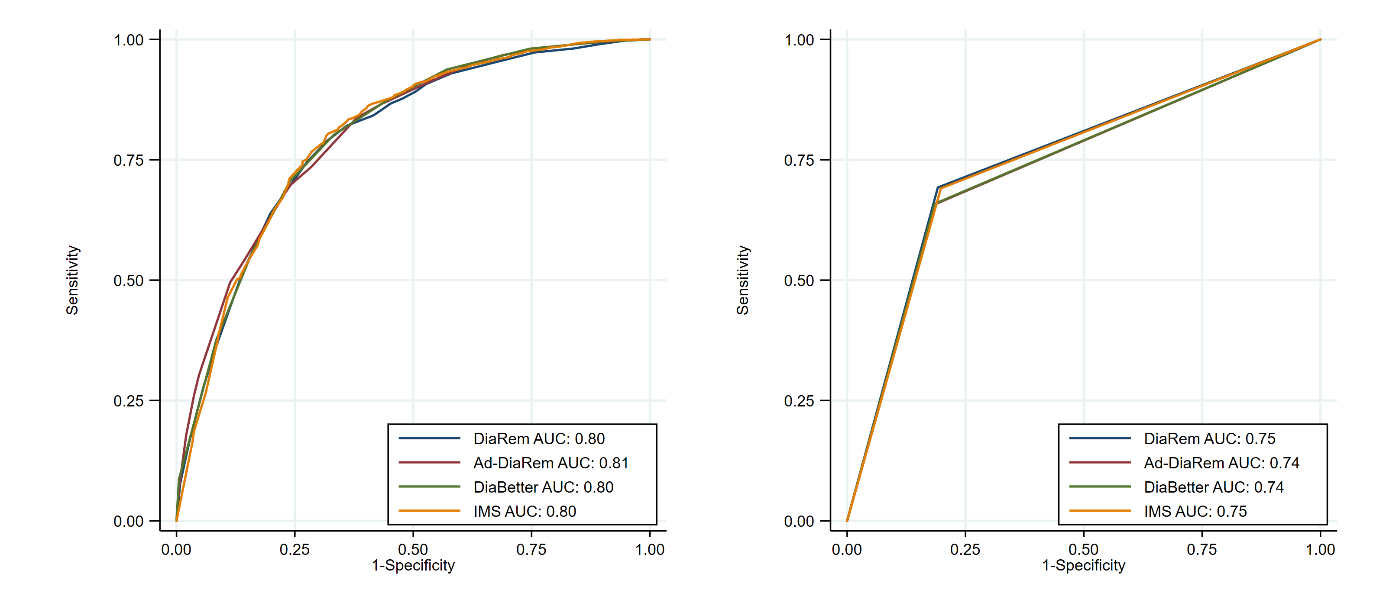


Supplementary Figure S3. ROC curves of diabetes indices in one of 100 trainings (left) and tests (right) for complete remission.

Supplementary Figure S4. A sequentially model training process


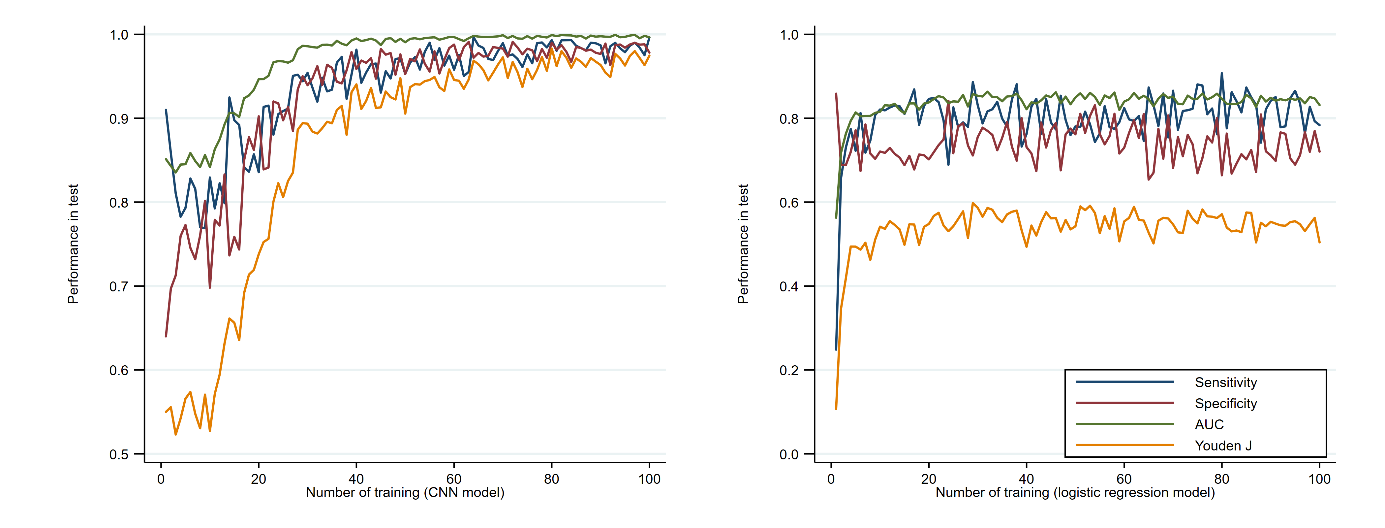


Supplementary Figure S5. Predictive ability of the CNN model (left) and the logistic regression model (right) in the test dataset using the continuously training process
